# Pathogenic potential of antibodies to the GABA_B_ receptor

**DOI:** 10.1002/epi4.12067

**Published:** 2017-07-19

**Authors:** Anjan Nibber, Edward O. Mann, Philippa Pettingill, Patrick Waters, Sarosh R. Irani, Dimitri M. Kullmann, Angela Vincent, Bethan Lang

**Affiliations:** ^1^ Nuffield Department of Clinical Neurosciences University of Oxford John Radcliffe Hospital Oxford United Kingdom; ^2^ Department of Physiology, Anatomy and Genetics University of Oxford Oxford United Kingdom; ^3^ Institute of Neurology University College London London United Kingdom

**Keywords:** Autoantibodies, Autoimmune encephalitis, Epilepsy, GABA_B_R, Whole‐cell recordings

## Abstract

GABA_B_ receptor (GABA_B_R) autoantibodies have been detected in the serum of immunotherapy‐responsive patients with autoimmune encephalitis. This study aimed to investigate the effect of immunoglobulin G (IgG) from a patient with GABA_B_R antibodies on primary neuronal cultures and acute slices of entorhinal cortex. Primary hippocampal neuronal cultures were incubated with serum immunoglobulin from patients with GABA_B_R or AMPA receptor (AMPAR) antibodies for up to 72 h to investigate their effect on receptor surface expression. Whole‐cell patch‐clamp recordings from layer III pyramidal cells of the medial entorhinal cortex were used to examine the effect on neuronal activity. GABA_B_R surface expression was unaltered by incubation with GABA_B_R antibodies. By contrast, after 24 h application of AMPAR antibodies, AMPARs were undetectable. However, acute application of GABA_B_R IgG decreased both the duration of network UP states and the spike rate of pyramidal cells in the entorhinal cortex. GABA_B_R antibodies do not appear to affect GABA_B_Rs by internalization but rather reduce excitability on the medial temporal lobe networks. This unusual mechanism of action may be exploited in rational drug development strategies.

Autoantibodies (Abs) to neuronal surface proteins comprise an expanding group of immunoresponsive central nervous system (CNS) diseases.^1^ The pathogenic effects of these Abs in vitro are usually considered to be mediated by Ab‐induced internalization of the antigenic target.^2^

Abs directed against the GABA_B_ receptor (GABA_B_R) were originally described in patients with limbic encephalitis (LE) who presented with early seizures.^3^ Follow‐up studies have shown an expanded phenotype, which includes ataxia, opsoclonus‐myoclonus syndrome, status epilepticus, and Lambert‐Eaton myasthenic syndrome (LEMS), usually in the context of encephalitis.^4^, ^5^, ^6^ Some GABA_B_R‐Ab‐positive patients have responded well to immunotherapies; however, many have a poor prognosis. Therefore, effective treatment of GABA_B_R‐mediated encephalitis remains an unmet medical need.

Higher cognitive functions rely on persistent cortical activity, and dysregulation in cortical networks may result in neurological impairment. During sleep and quiet wakefulness, cortical networks display intrinsic oscillations between active UP states and quiescent DOWN states. In the medial entorhinal cortex (mEC), these UP/DOWN states are maintained in vitro^7^ and are partly regulated by GABA receptors.^8^ Application of the GABA_B_R antagonist CGP55845 resulted in increased UP state duration, suggesting tonic GABA_B_R activity contributes to the spontaneous termination of UP states.

The aim of this study was to investigate the effects of GABA_B_R‐Abs on the surface expression of GABA_B_Rs in primary neuronal cultures and their impact on activity in acute entorhinal cortex (EC) slices using whole‐cell patch‐clamp recordings.

## Methods

### Cell‐based assays and internalization experiments

Patients were chosen for whom we had a sufficient quantity of serum with high levels of GABA_B_R or AMPA receptor (AMPAR) Abs. The research was approved by the Oxfordshire Research Ethics Committee A (07 Q160X/28 and 07 Q1604/28). Immunoglobulin G (IgG) was purified from a GABA_B_R‐ and an AMPAR‐Ab‐positive patient as previously described^9^ and shown to bind to GABA_B_Rs or AMPARs in cell‐based assays (CBAs). Briefly, human embryonic kidney (HEK) cells were transiently transfected with plasmids encoding either GABA_B_R‐1 and GABA_B_R‐2 or the AMPAR‐1 and AMPAR‐2 subunits. To assess effects of antibodies on GABA_B_R and AMPAR surface expression, neuronal cultures were exposed to the patient IgG (100 μg/mL; 1:100) for 1 or 24 h, followed by incubation with anti‐human IgG Alexa Fluor 488 secondary Ab, and visualized by fluorescence microscopy.^10^

### Electrophysiology and data analysis

Postnatal day 8–14 B57BL/6 mice were anesthetized using isoflurane (4%–5%) and decapitated, according to British Home Office regulations. Brains were removed and placed in ice‐cold artificial cerebrospinal fluid (aCSF; 126 mm NaCl, 3 mm KCl, 1.25 NaH_2_PO_4_, 1 mm MgSO_4_, 1 mm CaCl_2_, 24 mm NaHCO_3_, and 10 mm glucose, pH 7.2–7.4), containing 3 mm kynurenic acid. Horizontal brain sections, 350 μm thick, containing the EC and hippocampus were cut using a Leica VT1200S vibratome and incubated in an interface chamber between humidified carbogen gas (95% O_2_ and 5% CO_2_) and aCSF at room temperature for at least 1 h prior to recording.

### Electrophysiology and cell recordings

Slices were mounted on glass coverslips (coated with 0.1% poly‐L‐lysine) and placed in the recording chambers. Slices were superfused with aCSF (bubbled with carbogen), and the chamber maintained at a constant temperature (31°–33°C). Whole‐cell current‐clamp recordings were obtained from layer III mEC pyramidal neurons using borosillicate glass pipettes (5–8 MΩ) filled with internal solution containing 110 mm potassium gluconate, 40 mm HEPES, 2 mm Mg‐ATP, 0.3 mm NaGTP, and 4 mm NaCl (adjusted to pH 7.2–7.3 with KOH). Signals were low‐pass‐filtered at 2 kHz, acquired at 10 kHz using a Multiclamp 700B amplifier (Molecular Devices), and digitized using an ITC‐18 A/D board (Instrutech). Stimulation and recordings were controlled using previously described custom‐written procedures^8^ in Igor Pro (Wavemetrics). UP states were evoked every 30 s via a stimulation electrode placed in layer III (LIII) approximately 200 μm away from the recorded cell (stimulation strength: 100–200 μA, 100–200 μs). Current was increased until reliable UP states were evoked. Spike rate was defined as the spikes occurring during an UP state event and reaching a threshold potential of −20 mV. UP states were recorded from the mEC and compared in three conditions (at a flow rate of 5 mL/min); baseline aCSF, IgG diluted in aCSF and applied to brain sections at a concentration of 100 μg/mL, and a final wash step. UP states were recorded for a minimum of 10 min in each condition.

Statistical comparisons were made using repeated measures ANOVA, and Dunn's multiple comparison post hoc p < 0.05 was considered significant. All data are presented as mean ±SEM.

## Results

### Patient selection and clinical data

IgG was purified from a 50‐year‐old male patient who presented with confusion, unsteadiness, and proximal leg weakness. Following respiratory failure, he was admitted to the intensive care unit, and electroencephalogram recordings revealed a diffuse encephalopathy; electromyography was consistent with LEMS. Chest CT revealed a small‐cell lung carcinoma. The patient was treated with plasma exchange and underwent chemotherapy for his tumor, with good response. The patient had low serum levels of voltage‐gated calcium channel (VGCC)‐Abs (57 pm, normal <50 pm), but VGCC‐Ab levels in the IgG, purified from the plasma exchange eluate, were undetectable. GABA_B_R‐Ab levels were high (end‐point titration 1:1,000). IgG was also prepared from an AMPAR‐Ab patient (end‐point titration 1:1,000) and from a healthy individual (HC, 43‐year‐old male), with no prior neurological or autoimmune condition history, and whose serum and IgG tested negative on all diagnostic CBAs.

**Figure 1:**
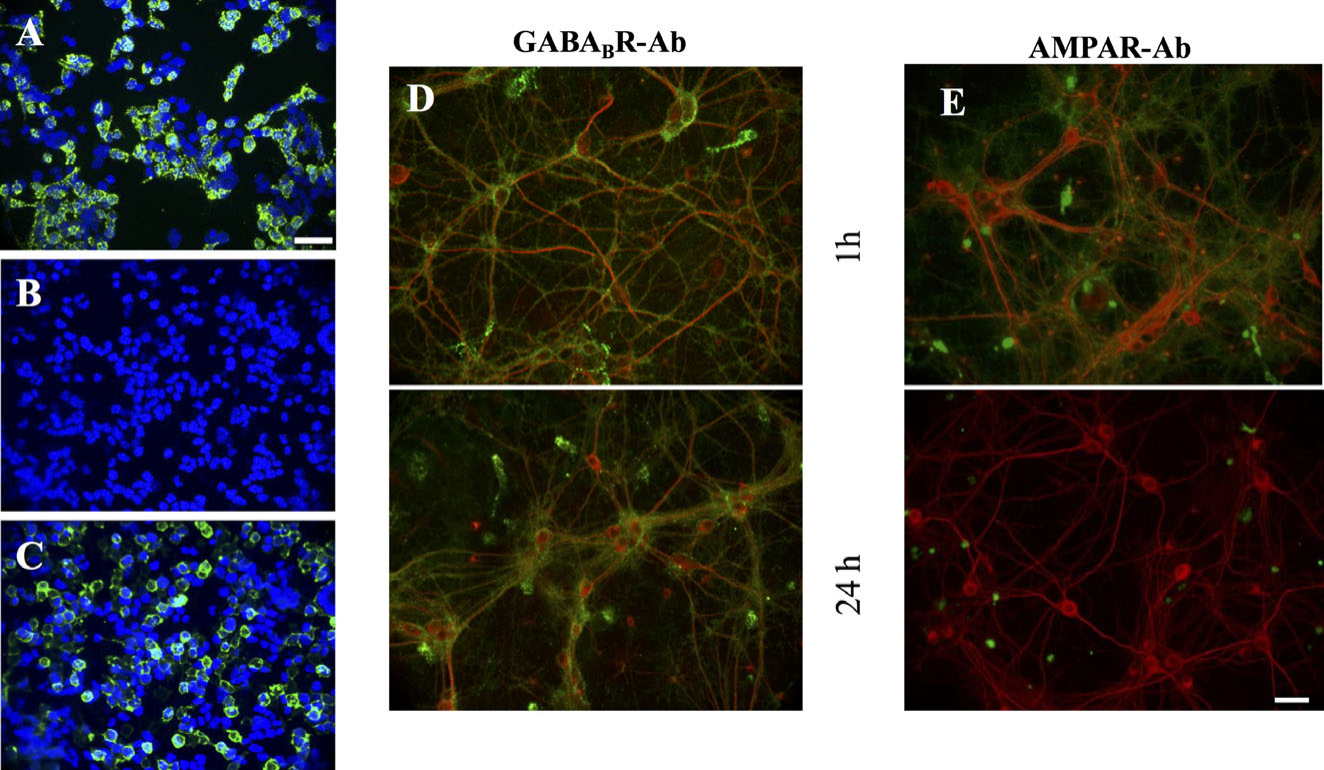
Expression studies of GABA_B_R on transfected cells and neuronal cultures. Immunostaining of HEK cells expressing GABA_B_R; 1:1 ratio, GABA_B_R1 and GABA_B_R2 subunits (**A**) and HC serum (**B**) and compared to AMPAR‐Ab‐positive patient on HEK cells expressing 1:1 ratio of AMPAR1 and AMPAR2: (**C**), dilution 1:100 in all cases, staining visualized with goat anti‐human IgG, Alexa Fluor 488 (green), counterstained with DAPI (blue). Scale bar represents 50 μm. Cultured hippocampal neurons (div 14) were incubated with patient sera (1:100) for 1 h, and staining was visualized with goat anti‐human IgG Alexa Fluor 488 (green). Neurons were subsequently fixed, permeabilized, and incubated with microtubule‐associated protein 2 (MAP2; Alexa Fluor 568 (red)). Merged images are shown. GABA_B_R‐Ab IgG (green) showed similar reactivity to neuronal cultures following 1 or 24 h incubation (**D**), suggesting surface expression is not affected. In contrast, AMPAR‐Ab IgG staining was lost at 24 h (**E**). Scale bar represents 100 μm.

IgG (100 μg/mL) purified from the GABA_B_R‐Ab‐positive patient showed strong immunoreactivity to the extracellular domains of GABA_B_R by CBA (Fig. 1A) and strong binding to neuronal cultures after 1 h incubation, which was retained at 24 h (Fig. 1D). In contrast, after incubation with IgG (100 μg/mL) purified from a patient with AMPAR‐Abs, AMPAR expression was retained on the surface of primary neuronal cultures at 1 h, but lost after 24 h incubation (Fig. 1E).

### Electrophysiology

#### Effect of IgG on UP state duration

**Figure 2:**
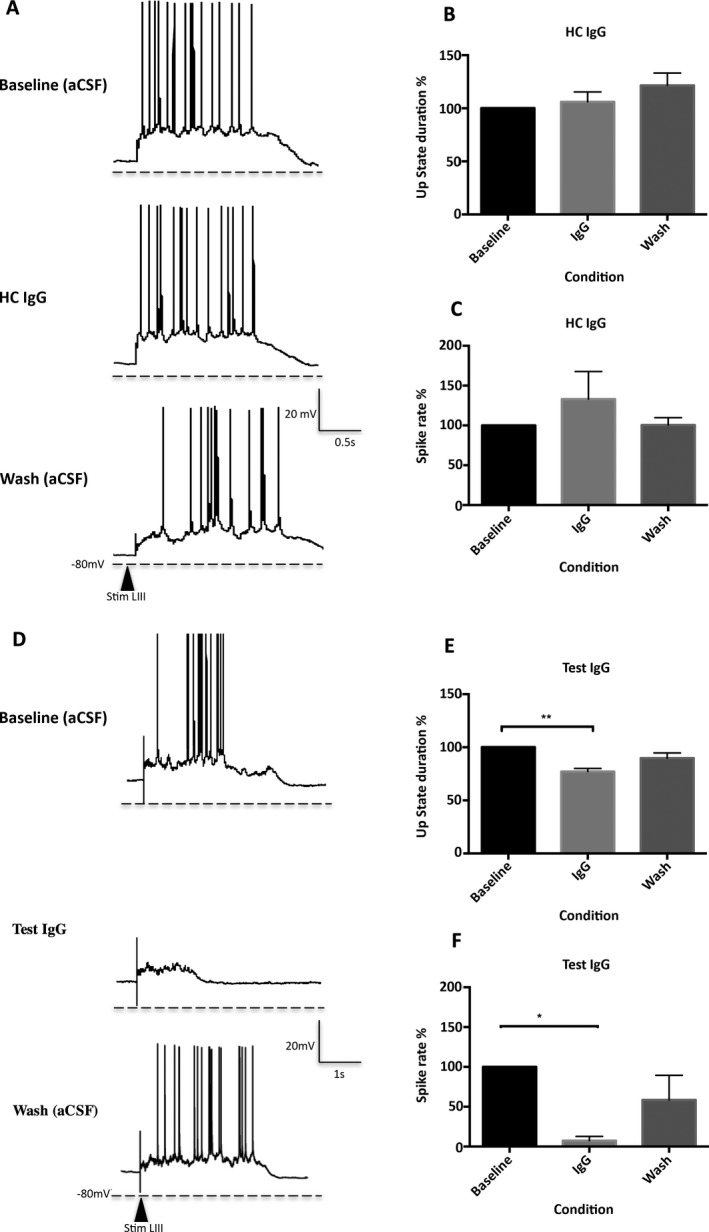
The effect of IgG on UP state duration and spike rate. (**A–C)** Effect of HC IgG (100 μg/mL) on UP states. Representative image showing UP state duration and spike rate are unaffected following the application of HC IgG. Top panel: UP state recordings taken during baseline recordings in aCSF. Middle panel: HC IgG (100 μg/mL). Bottom panel: following aCSF wash. LIII; Layer III, Stim; Stimulation (**A**). Following application of HC IgG, UP state duration and spike rate were unaffected (pooled data from 4 slices) (**B** and **C**). (**D–F**) Effect of test IgG (GABA_B_R patient IgG) on UP states. Representative image showing both UP state duration and the spike rate were significantly reduced following the application of patient IgG. Top panel: UP state recordings taken during baseline recordings in aCSF. Middle panel: test IgG (100 μg/mL). Bottom panel: following aCSF wash. LIII; Layer III, Stim; Stimulation (**D**). UP state duration was significantly reduced following the application of test IgG (**p = 0.0028, pooled data n = 6) (**E**). Similarly, a significant reduction was noted in spikes present during UP state events following application of test IgG (*p = 0.0185, pooled data n = 5) (**F**).

UP states evoked by local synaptic stimulation in mEC were monitored using whole‐cell current‐clamp recordings from LIII pyramidal cells. No changes were noted when HC IgG (Figs. 2A–B) was applied to the slices, in comparison to baseline recordings or wash (baseline: 2.44 ± 0.45 s, HC IgG: 2.55 ± 0.41 s, wash: 2.81 ± 0.334 s, n = 5 slices from 4 mice, p = 0.23). Application of GABA_B_R‐Ab‐positive IgG (Figs. 2D–E) revealed a 23% reduction in UP state duration (baseline: 3.75 ± 0.38 s vs. test IgG: 2.90 ± 0.44 s, n = 6 slices, from 5 mice) in comparison to baseline recordings (p = 0.0028). Washing of cells recovered over 50% of the loss in UP state duration (wash: 3.35 ± 0.37 s), suggesting that the effects are partially reversible.

#### Effect of IgG on spike rate

Spike rate (mean spike count/mean UP state duration) was not significantly altered following the application of HC IgG on the slices (baseline: 1.81 ± 1.57 vs. HC IgG: 1.68 ± 1.08; pooled data shown in Fig. 2C, p = 0.9306). Following application of GABA_B_R‐Ab‐positive IgG to the slices, a 92% reduction in spike rate was noted (baseline: 1.22 ± 0.31 vs. test IgG: 0.10 ± 0.16, p = 0.0185; Fig. 2E). Although no statistical significance existed between the baseline and the wash recordings, a reduction was observed, perhaps indicative of a partial washout.

These effects were abolished after IgG preadsorption against HEK cells expressing GABA_B_R (data not shown), suggesting these are effects resulting from antigen‐specific modulation.

## Discussion

The binding of Abs to extracellular epitopes in patients with LE suggests these Abs are directly modulating epileptogenic neuronal networks. It has been demonstrated in vitro that Abs to the NMDA receptor or AMPAR down‐regulate surface receptors.^2^, ^11^ In contrast, the mechanism of action of GABA_B_R‐Abs remains unknown.

In this study, IgG purified from a GABA_B_R‐Ab positive patient did not internalize GABA_B_Rs. However, GABA_B_R‐Abs modulated network activity in mEC slices. Acute application of patient IgG caused a reduction in UP state duration and spike rates in LIII pyramidal cells measured by whole‐cell patch‐clamp recording. Synaptic inhibition plays an important role in regulating the excitability of cortical networks, and the GABA_B_R is important in regulating spontaneous GABA release in the EC,^12^ a region associated with temporal lobe epilepsies. Interference of this mEC circuitry may disrupt the system, reducing hypoexcitability, resulting in local epileptogenesis.

Our study provides the first evidence that GABA_B_R‐Abs have a direct modulatory effect on the function of GABA_B_R at CNS synapses. Because the study was limited to one patient by the large plasma volume required, future studies should examine the effects from a range of GABA_B_R‐Ab positive patients. The use of brain slices for whole‐cell recordings has numerous advantages but is an oversimplification of the in vivo situation, and animal models should be investigated to assess the pathogenic potential of these Abs.

## Disclosure of Conflict of Interest

P.W., A.V., S.R.I., B.L., and the Nuffield Department of Clinical Neurosciences in Oxford receive royalties and payments for antibody assays. P.W. has received speaker honoraria from Biogen Idec and Euroimmun AG and travel grants from the Guthy‐Jackson Charitable Foundation. The remaining authors have no conflicts of interest. We confirm that we have read the Journal's position on issues involved in ethical publication and affirm that this report is consistent with those guidelines.
